# FOXK2 downregulation suppresses EMT in hepatocellular carcinoma

**DOI:** 10.1515/med-2020-0129

**Published:** 2020-07-20

**Authors:** Jian Kong, Qingyun Zhang, Xuefeng Liang, Wenbing Sun

**Affiliations:** Department of Hepatobiliary Surgery, Beijing Chaoyang Hospital, Capital Medical University, Beijing 100043, China; Department of General Surgery, Affiliated Hospital of Chengde Medical University, Hebei 067000, China; Blood center of Shandong Province, Shandong 250000, China

**Keywords:** hepatocellular carcinoma, FOXK2, epithelial–mesenchymal transition, Akt

## Abstract

Forkhead box K2 (FOXK2) was first identified as an NFAT-like interleukin-binding factor. FOXK2 has been reported to act as either oncogene or tumor suppressor. However, functional and regulating mechanisms of FOXK2 in epithelial–mesenchymal transition (EMT) in hepatocellular carcinoma (HCC) remain unclear. An FOXK2-specific siRNA was employed to decrease the endogenous expression of FOXK2. MTT assay, colony formation and transwell assay were used to evaluate proliferation, migration and invasion of Hep3B and HCCLM3 cells, respectively. The protein expression associated with EMT and Akt signaling pathways was evaluated using western blot. FOXK2 downregulation could inhibit cell proliferation and colony formation and suppress migration and invasion in Hep3B and HCCLM3 cells. The expression of E-cadherin was significantly upregulated, and the expression of snail and p-Akt was significantly downregulated in siFOXK2-transfected cells compared with control cells. SF1670 induced the expression of p-Akt and snail and suppressed the expression of E-cadherin in Hep3B and HCCLM3 cells. SF1670 promoted the invasion and colony formation of Hep3B and HCCLM3 cells. SF1670 partly inhibited the effect of FOXK2 suppression on Hep3B and HCCLM3 cells. In conclusion, this study revealed that FOXK2 downregulation suppressed the EMT in HCC partly through inhibition of the Akt signaling pathway.

## 1 Introduction

Hepatocellular carcinoma (HCC) is listed as the sixth most common neoplasm and the third leading cause of cancer death, which has been recognized as a major cause of death among cirrhotic patients [1]. Surgical resection, liver transplantation and radiofrequency ablation are considered potentially curative for HCC [2]. However, most HCC patients are diagnosed at advanced stages when surgical treatments are unsuitable. Only sorafenib and regorafenib are proven to prolong survival in HCC [3,4,5,6,7,8]. Therefore, development of new targeted therapy for HCC treatment is urgent.

The structure of Forkhead box (FOX) proteins contains conserved DNA binding domain, which is defined as the forkhead winged helix-turn-helix. FOX proteins participate in various biological processes, which include metabolism, development, differentiation, apoptosis, proliferation, migration, invasion and longevity [9]. Forkhead box K2 (FOXK2), also regarded as an interleukin enhancer binding factor, was initially confirmed as a nuclear factor of an activated T cell-like interleukin-binding factor. In human tumors, FOXK2 has been reported to act as either oncogene or tumor suppressor [10]. In glioma, FOXK2 could inhibit the cell multiplication and motility and predict a favorable prognosis [11]. In clear-cell renal cell carcinoma, FOXK2 expression was downregulated in tumor tissue, which suppressed the capabilities of proliferation and motility and promoted apoptosis via suppression of epidermal growth factor receptor [12]. In breast cancer, FOXK2 could regress various genes containing HIF1β and EZH2, which further inhibited the hypoxia-induced response and tumor carcinogenesis [13]. In HCC, FOXK2 promoted cell growth and indicated unfavorable prognosis [14]. In ERα-positive breast cancer, FOXK2 could inhibit tumor growth via decreasing the ERα stability through BRCA1/BARD1-associated mechanisms [15]. In colorectal cancer, Sox9 could activate FOXK2 and further promote the tumor cell multiplication [16]. FOXK2 could translocate DVL into the nucleus and further activate the Wnt/β-catenin signaling pathway [17]. In breast cancer, FOXK2 could also mediate epirubicin and paclitaxel resistance via FOXO3a [18]. However, the concrete mechanism of FOXK2 in HCC is still unclear.

High rates of tumor recurrence and metastasis lead to poor prognosis in patients with advanced HCC [19]. Metastasis is a complicated biological process, which is involved in a poly-stage cascade of genetic and epigenetic events [20]. Epithelial–mesenchymal transition (EMT) plays a crucial role in tumor metastasis. During the process of EMT, polarized epithelial cells transform into fibroblast-like mesenchymal cells, and the cells become more motile and invasive and more susceptible to metastasis [21,22,23,24]. Transforming growth factor-β (TGF-β), which is increasing in cirrhosis and late-stage HCC, could induce EMT in HCC [25]. FOXK2 could inhibit the EMT of non-small cell lung cancer (NSCLC) [26]. Whether FOXK2 is involved in the process of EMT in HCC remains uncertain.

In the present study, we evaluated the biological functions of FOXK2 using HCC cell line and the effect of FOXK2 knockdown on EMT, the malignant phenotype and associated signaling pathways. We demonstrated that FOXK2 downregulation suppressed the EMT in HCC cell lines through inhibition of the Akt pathway.

## 2 Materials and methods

### 2.1 Cell lines and cell culture

Hep3B and HCCLM3 cells were obtained from the Cell Bank of the Chinese Academy of Sciences (Shanghai, China). The cells were cultured in Dulbecco’s modified Eagle’s medium (DMEM; Gibco, USA) supplemented with 10% fetal bovine serum (FBS; Gibco, USA) and incubated in 5% CO_2_ at 37°C.

### 2.2 Cell proliferation assay

Cell proliferation was assessed using the MTT method. Briefly, cells were seeded at 3 × 10^3^ cells/well into a 96-well plate and were cultured for 24, 48 or 72 h. In the end of the incubation, MTT (Sigma-Aldrich, USA) solution was added to each well at a final concentration of 5 mg/mL and incubated at 37°C for 4 h. Dimethyl sulfoxide (150 µL) was used to dissolve the formazan crystals. The absorbance was detected at 570 nm using a microplate reader (Bio-Rad Laboratories, USA).

### 2.3 Colony formation assay

Briefly, Hep3B or HCCLM3 cells with or without FOXK2 knockdown were trypsinized, and 1 × 10^3^ cells/well were added into six-well plates. The cells were continually cultured at 37°C for 14 days. Wells were stained with 0.1% crystal violet (Solarbio, China) for 20 min before counting colonies under a dissecting microscope.

### 2.4 Migration and invasion assays

After FOXK2 knockdown, Hep3B or HCCLM3 cells were collected, and 2 × 10^4^ cells or 5 × 10^4^ cells in the medium were added to the upper chamber of transwell plates (Corning, USA) with or without BD Matrigel (BD Biosciences, USA) matrix coating. Medium containing 10% FBS was added into the lower chambers. After 24 h culture, the non-migrating or non-invading cells in the upper chamber were gently removed, and the migrating or invading cells were stained with 0.1% crystal violet (Solarbio, China) for 20 min, imaged and counted under a microscope.

### 2.5 Gene transfection

Hep3B or HCCLM3 cells were cultured in six-well plates at a density of 5 × 10^5^ cells/well. The gene transfection assay was performed with the following constructs using Lipofectamine 2000 (Invitrogen, USA) according to the manufacturer’s instructions. FOXK2 siRNA sequences were as follows: 5′-GCGAGUUCGAGUAUCUGAUTT-3′ (sense) and 5′-AUCAGAUACUCGAACUCGCTT-3′ (antisense). Negative control siRNA sequences were as follows: 5′-UUCUCCGAACGUGUCACGUTT-3′ (sense) and 5′-ACGUGACACGUUCGGAGAATT-3′ (antisense). Cells were cultured with siRNA in DMEM for 6 h, then the medium was replaced with DMEM containing 10% FBS. The cells were incubated for 48 h and collected for next experiments. The concentration of siRNA was 100 nmol/L.

### 2.6 Western blot analysis

Total proteins were extracted using RIPA containing protease and phosphatase inhibitors. Protein concentrations were measured by the bicinchoninic acid method. The protein samples (50 µg) were separated by SDS-PAGE and transferred onto nitrocellulose membranes (EMD Millipore, USA). Immunoblotting was performed with antibodies against β-actin (1:2,000), E-cadherin (1:1,000), p-Akt (1:500), t-Akt (1:1,000), snail (1:500) (Abcam, Cambridge, MA, USA) and FOXK2 (1:500; CST, Boston, MA, USA). Then, the blot was examined with HRP-labeled secondary antibody (1:4,000). The visualization of protein bands was detected using the ECL detection system (Millipore, USA) according to the manufacturer’s instructions.

### 2.7 Statistical analysis

All data are presented as mean ± SD. GraphPad Prism8 (GraphPad Software, La Jolla, CA, USA) was used for statistical analysis. Comparison between two or multiple groups was performed using Student’s *t*-test or one-way analysis of variation. *P* < 0.05 was considered statistically significant.

## 3 Results

### 3.1 FOXK2 downregulation inhibits cell growth and colony formation in Hep3B or HCCLM3 cells

**Figure 1:**
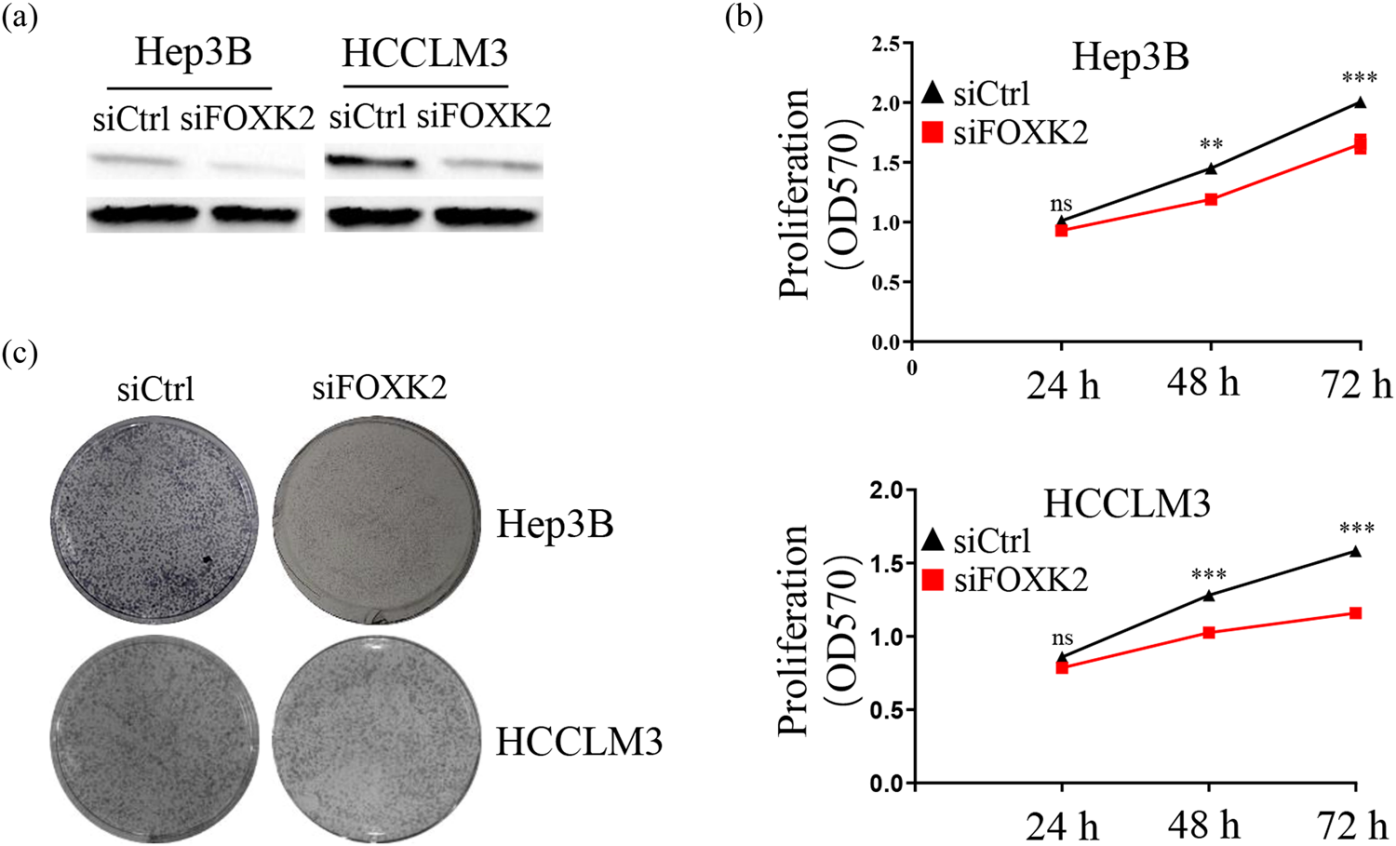
FOXK2 downregulation inhibits cell growth and colony formation in Hep3B and HCCLM3 cells. The expression of FOXK2 was knocked down in Hep3B or HCCLM3 cells to define the biological effect of FOXK2. (a) The efficiency of knockdown of FOXK2 was confirmed by western blot. (b) FOXK2 knockdown dramatically inhibited cell proliferation of Hep3B or HCCLM3 cells at 48 and 72 h. (c) FOXK2 knockdown also dramatically inhibited the colony formation of Hep3B or HCCLM3 cells. These data are representative of at least three independent experiments. ns: no significance; ^**^ *P* < 0.01 and ^***^ *P* < 0.001.

To determine the biological effect of FOXK2 on HCC, the FOXK2 expression was knocked down in Hep3B or HCCLM3 cells. The efficiency of FOXK2 knockdown was confirmed by western blot (Figure 1a). We analyzed the influence of FOXK2 on cell proliferation. FOXK2 knockdown dramatically inhibited cell proliferation of Hep3B or HCCLM3 cells at 48 and 72 h (Figure 1b). Meanwhile, FOXK2 knockdown also dramatically inhibited the colony formation of Hep3B or HCCLM3 cells (Figure 1c). Similar results were also found using another FOXK2 siRNA (Supplementary Figure 1).

### 3.2 FOXK2 knockdown suppresses the migration and invasion of Hep3B or HCCLM3 cells

**Figure 2:**
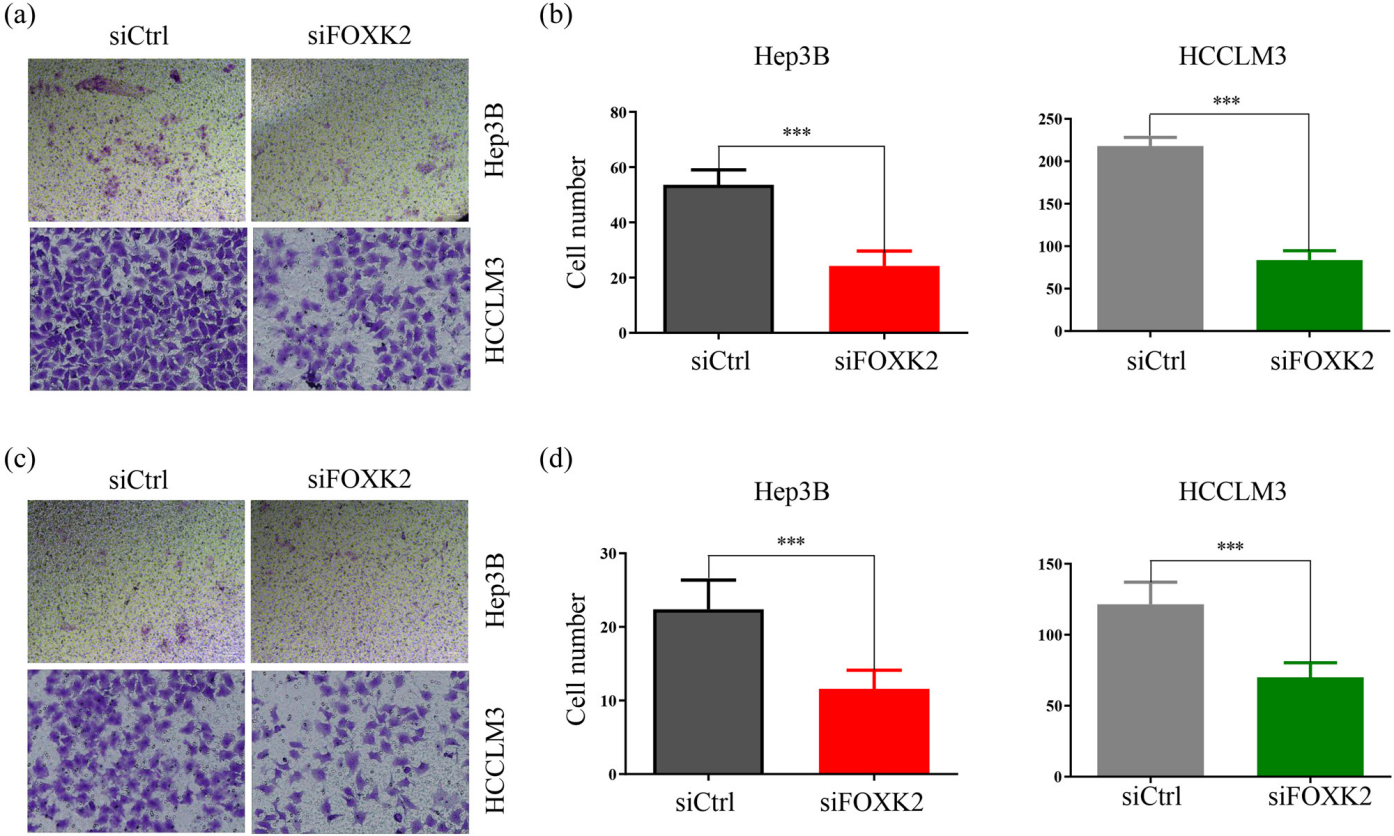
FOXK2 knockdown suppresses the migration and invasion of Hep3B or HCCLM3 cells. We explored the role of FOXK2 in the migration and invasion of Hep3B or HCCLM3 cells. (a and b) Migration assay demonstrated that cell motility was significantly decreased in siFOXK2-transfected cells compared with control cells. (c and d) The matrigel invasion assay showed that invasiveness was significantly suppressed in siFOXK2-transfected cells. These data are representative of at least three independent experiments. ^***^ *P* < 0.001.

We also explored the effect of FOXK2 on the migration and invasion of Hep3B or HCCLM3 cells. Migration assay suggested that migrated cell numbers in siFOXK2-transfected cells were significantly less than that of the control group in Hep3B and HCCLM3 cells, which demonstrated that cell motility was significantly decreased after FOXK2 knockdown (Figure 2a and b). Similarly, the matrigel invasion assay demonstrated that invasiveness was significantly suppressed in siFOXK2-transfected cells (Figure 2c and d).

### 3.3 FOXK2 may regulate the EMT in Hep3B or HCCLM3 cells through Akt signaling

**Figure 3:**
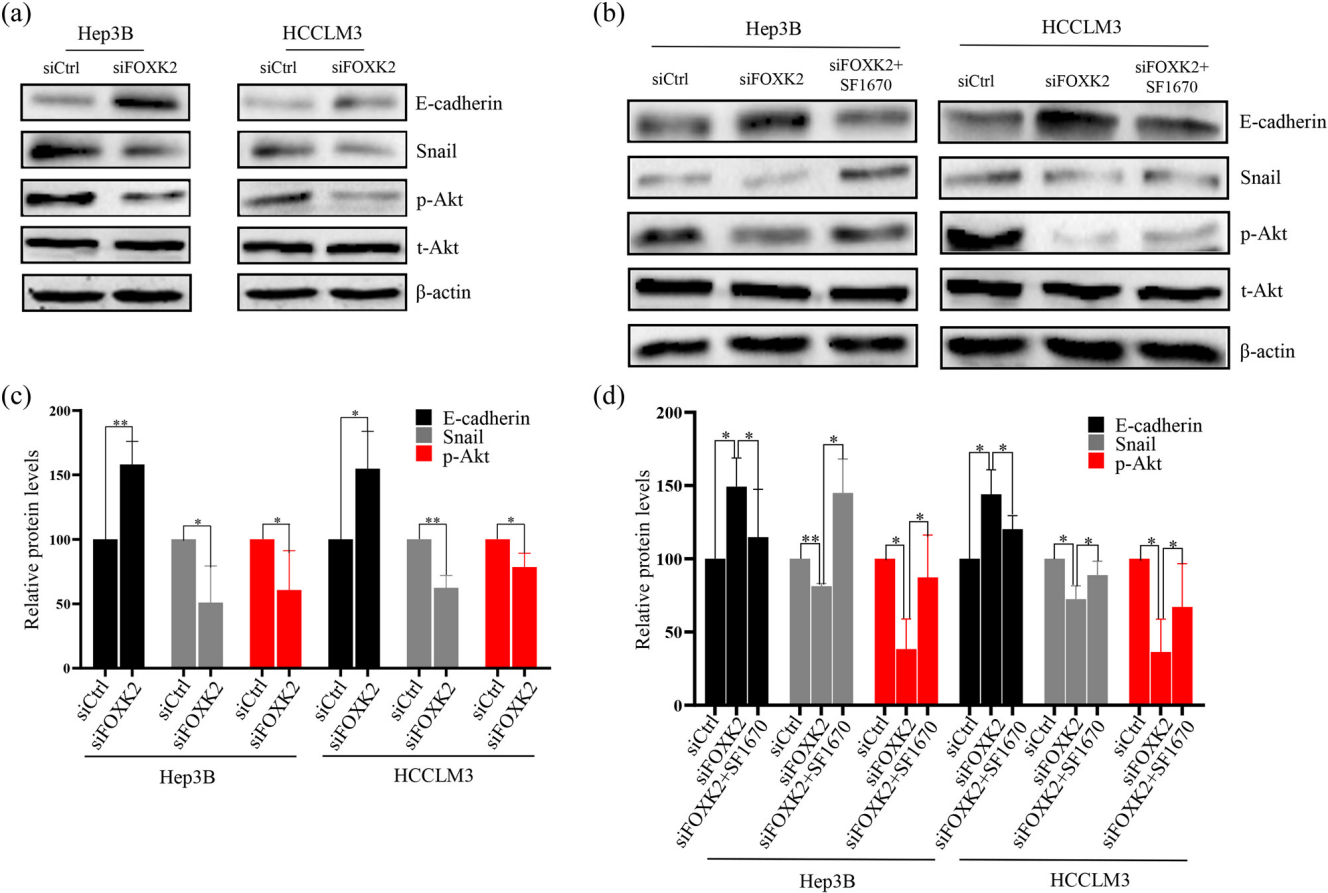
FOXK2 may regulate the EMT in Hep3B or HCCLM3 cells through the Akt signaling pathway. We analyzed the influence of FOXK2 on EMT markers and related signals. (a and b) E-cadherin expression was significantly upregulated and snail and p-Akt expression was significantly downregulated in siFOXK2-transfected cells compared with control cells. (c and d) To confirm whether the Akt pathway participated in the process of FOXK2 regulating the EMT in Hep3B cells, SF1670 was used to activate PI3K/Akt, which is a PTEN-specific inhibitor. SF1670 upregulated the expression of p-Akt and snail and downregulated the expression of E-cadherin. These data are representative of three independent experiments. ^*^ *P* < 0.05 and ^**^ *P* < 0.01.

To verify the associated mechanism of FOXK2 involved in the biological changes in Hep3B or HCCLM3 cells, we also observed the role of FOXK2 in EMT markers and related signals. E-cadherin expression was significantly upregulated, and snail and p-Akt expression was significantly downregulated in siFOXK2-transfected cells compared with control cells (Figure 3a and b). To confirm whether the Akt signaling pathway was involved in the process of FOXK2 regulating the EMT in Hep3B or HCCLM3 cells, SF1670 was used to activate PI3K/Akt, which is a PTEN-specific inhibitor. SF1670 increased the expression of p-Akt and snail and decreased the expression of E-cadherin (Figure 3c and d).

**Figure 4:**
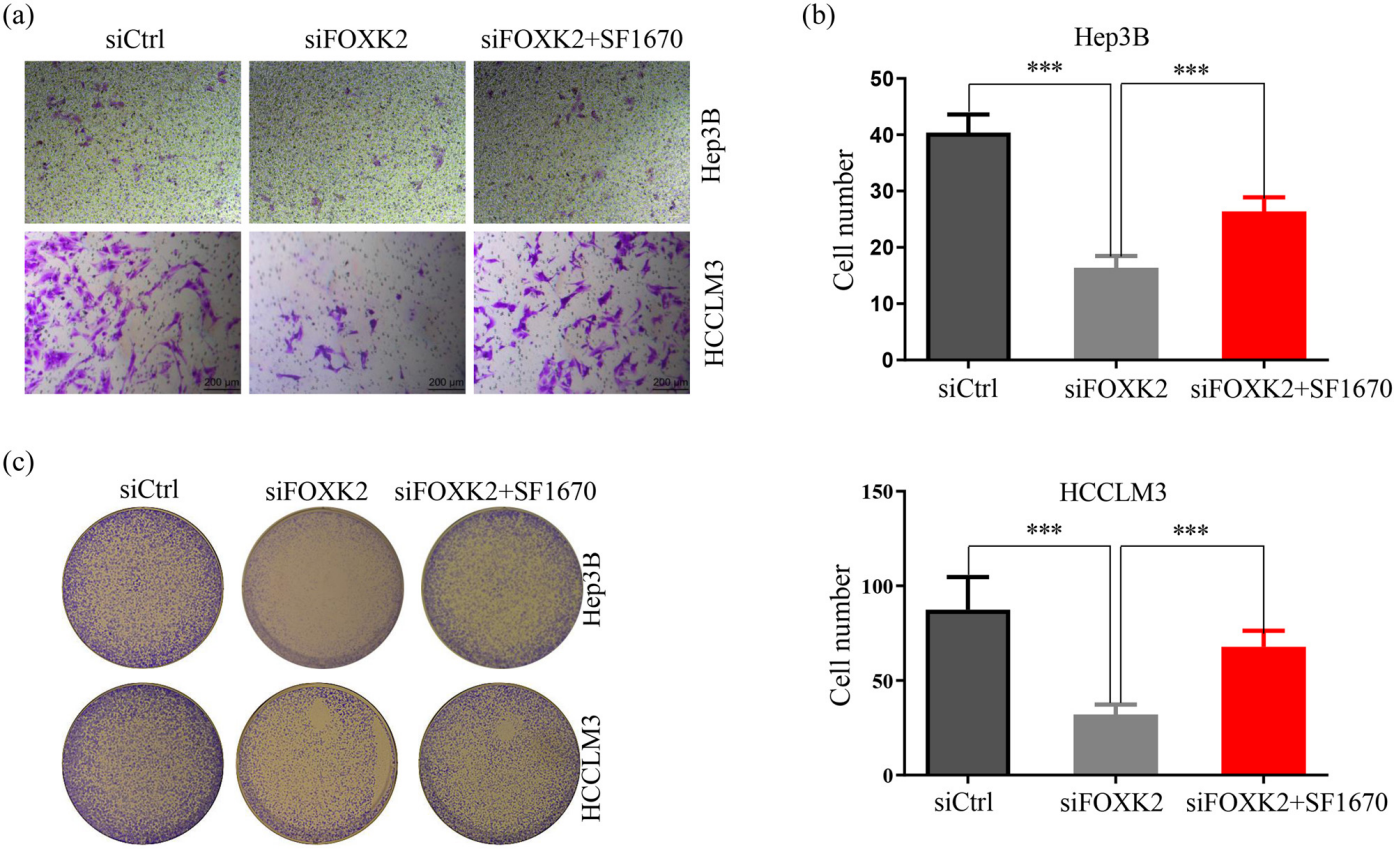
Akt activation blocked the effect of FOKK2 inhibition on HCC cells. FOXK2 knockdown dramatically inhibited the invasion (a and b) and colony formation (c) of Hep3B or HCCLM3 cells and SF1670 partly inhibited the effect of FOXK2 suppression on HCC cells. These data are representative of at least three independent experiments. ^***^ *P* < 0.001.

We also observed the effect of SF1670 on FOXK2 regulating the biological changes in Hep3B or HCCLM3 cells. SF1670 promoted the invasion and colony formation of HCC cells and partly inhibited the effect of FOXK2 inhibition on HCC cells (Figure 4).

## 4 Discussion

FOXK2 has been reported to act as either oncogene or tumor suppressor; however, the effect of FOXK2 on the EMT has not yet been demonstrated in HCC cells. In the current research, we demonstrated that FOXK2 knockdown inhibited the proliferation, colony formation, migration and invasion of HCC cells. Furthermore, we describe a possible mechanism by which FOXK2 downregulation inhibited EMT. In particular, FOXK2 downregulation suppressed the EMT of HCC cells partly via inhibition of the Akt pathway.

FOXK2 functions as oncogene in HCC [27] and colorectal cancer or tumor suppressor in NSCLC, glioma, breast cancer and renal cell carcinoma [12,28]. FOXK2 also regulated the sensitivity of breast cancer to epirubicin and paclitaxel. FOXK2 could suppress cell proliferation and invasion in clear-cell renal cell carcinoma and breast cancer and inhibit tumor growth in breast cancer. However, FOXK2 was obviously increased in HCC and associated with tumor size, TNM stage and tumor vascular invasion, and FOXK2 upregulation in HCC cells could lead to increased cell proliferation and migration [27]. Our results suggested that FOXK2 also functioned as oncogene and loss of FOXK2 inhibited cell proliferation, migration and invasion in HCC. FOXK2 might be involved in HCC carcinogenesis and function as a tumor promoter through suppression of cell proliferation and migration.

EMT facilitates cancer cell invasion and results in tumor metastasis. EMT could be activated by extracellular proteases, which promoted EMT-associated proteins. TGF-β1 is a key factor in the process of EMT, and E-cadherin on the cell surface is cleaved, which disrupts E-cadherin-mediated cell–cell adhesion and results in EMT [29]. Snail acts as a crucial transcription factor that plays a role as a suppresser of E-cadherin expression and an inducer of EMT in different kinds of cancer [30]. Previous research studies have revealed that the FOXK2 suppressed EMT in NSCLC and glioma via suppression of various pivotal target genes [11,26]. In this study, we showed that FOXK2 knockdown could increase the E-cadherin expression and decrease the Snail expression, which suggested that FOXK2 could promote EMT in HCC cells.

FOXK2 could prevent tumor progression via the p53 pathway, hypoxia pathway or β-catenin signaling pathway [13,17]. Furthermore, FOXK2 could also cause oncogenic activity in HCC through the PI3K/Akt signaling pathway. The PI3K/Akt signaling pathway has been reported to be involved in the EMT of HCC [31,32,33,34,35]. The PI3K/Akt signaling pathway might play an important role in the FOXK2 regulating EMT in HCC. This study showed that FOXK2 knockdown could decrease the expression of p-Akt, and the activation of PI3K/Akt could reverse the process of EMT, which suggested that the PI3K/Akt signaling pathway played a crucial role in the FOXK2 regulating EMT in HCC.

In conclusion, this work showed that FOXK2 downregulation suppressed the EMT progress partly through inhibition of the PI3K/Akt pathway in HCC cells. These findings have important implications for understanding the mechanisms and may provide a new therapeutic target for HCC treatment.
